# Restriction Endonucleases that Bridge and Excise Two Recognition Sites from DNA

**DOI:** 10.1016/j.jmb.2006.12.070

**Published:** 2007-03-23

**Authors:** Jacqueline J.T. Marshall, Darren M. Gowers, Stephen E. Halford

**Affiliations:** The DNA–Protein Interactions Unit, Department of Biochemistry, School of Medical Sciences, University of Bristol, University Walk, Bristol BS8 1TD, UK

**Keywords:** AdoMet, *S*-adenosyl methionine, KOAc, Mg(OAc)_2_ and Tris-OAc, potassium, magnesium and Tris acetate, respectively, R-M, restriction–modification, U, units of restriction enzyme activity, FLL, full-length linear, OC, open-circle, SC, supercoiled, restriction enzyme, DNA–protein interaction, enzyme mechanism, DNA excision, DNA looping

## Abstract

Most restriction endonucleases bridge two target sites before cleaving DNA: examples include all of the translocating Type I and Type III systems, and many Type II nucleases acting at their sites. A subset of Type II enzymes, the IIB systems, recognise bipartite sequences, like Type I sites, but cut specified phosphodiester bonds near their sites, like Type IIS enzymes. However, they make two double-strand breaks, one either side of the site, to release the recognition sequence on a short DNA fragment; 34 bp long in the case of the archetype, BcgI. It has been suggested that BcgI needs to interact with two recognition sites to cleave DNA but whether this is a general requirement for Type IIB enzymes had yet to be established. Ten Type IIB nucleases were tested against DNA substrates with one or two copies of the requisite sequences. With one exception, they all bridged two sites before cutting the DNA, usually in concerted reactions at both sites. The sites were ideally positioned *in cis* rather than *in trans* and were bridged through 3-D space, like Type II enzymes, rather than along the 1-D contour of the DNA, as seen with Type I enzymes. The standard mode of action for the restriction enzymes that excise their recognition sites from DNA thus involves concurrent action at two DNA sites.

## Introduction

Restriction–modification (R-M) systems can be classified into four main groups on the basis of their genetic and polypeptide organisations, their modes of action and their co-factor requirements: Types I, II, III and IV.^1,2^ Most bacterial genomes encode multiple R-M systems: about 35% of the total are Type I systems, 40% Type II, 10% Type III and 15% Type IV.^3^ The restriction enzymes from the Type I and the Type III systems are ATP-dependent proteins that initiate DNA translocation at specific recognition sites and then cleave DNA some distance from the sites, usually upon collision of two complexes translocating from separate sites.^4,5^ Type I restriction enzymes normally require two recognition sites *in cis* to cleave DNA, though they can cleave circular DNA with one site, while the Type III nucleases almost always need two sites.^6,7^ The Type IV systems are GTP-dependent enzymes that restrict DNA that has been methylated at two or more sites.^5,8^ The endonucleases from the Type I, III and IV families thus constitute ∼60% of the restriction enzymes found in nature, and they all interact with two target sites before cleaving DNA.^9^

While the Type I and Type III systems feature multi-subunit proteins with both endonuclease and methyltransferase activities,^4,5^ Type II R-M systems generally comprise two separate proteins, one for each activity,^10^ though in some cases both functions are fused into a single polypeptide.^11,12^ Unlike the NTP-dependent systems, the Type II endonucleases cleave DNA at fixed positions in or near their recognition sites.^3^ Most (but not all)^13^ need Mg^2+^ as a cofactor,^10,14^ though some of the fused proteins additionally need the methyl donor *S*-adenosyl methionine (AdoMet) not only for methyltransferase but also for nuclease activity.^11,12^ The standard Type II restriction enzymes, such as EcoRV and BamHI, are homodimers that recognise palindromic sequences, typically 4–8 bp long, and cut both strands at specified positions within the sequence.^14,15^ They cleave DNA with multiple sites by means of separate reactions at each site.^16–18^

Of the ∼4000 Type II restriction enzymes identified to date^3^ many differ from the standard and have been classified into various sub-types.^2^ One tenet of this classification is their mode of action at multiple recognition sites. Numerous Type II enzymes, maybe 50% of the total,^9,18^ cannot cleave DNA without first interacting with two copies of their sites, either looping out the DNA between two sites *in cis* on the same chain of DNA, or bridging sites *in trans* on separate molecules of DNA.^19–23^ The enzymes that need two sites can be identified by comparing their activities on DNA substrates with one or two copies of the requisite sequence.^24,25^ Under most circumstances, an enzyme that binds two DNA loci has a higher affinity for sites in the same chain over sites in separate molecules,^9^ so will usually cleave two-site DNA more rapidly than one-site DNA.^24^ However, an enzyme that needs two sites but which has a high affinity for its site may have sufficient affinity for sites *in trans* to give its *V*_max_ on a DNA with one site.^25^ Nevertheless, DNA–protein interactions are weakened at elevated ionic strengths so even if an enzyme gives equal rates on the one and two-site substrates at low ionic strength, it may cleave the two-site DNA faster than the one-site DNA at raised ionic strength.^16,18,26^ Alternatively, these enzymes can be identified from whether they can be activated to cleave plasmids with one target site by adding oligonucleotide duplexes that also carry the target sequence, as a result of interactions *in trans* between plasmid and oligoduplex.^20,25,27^

The Type II restriction enzymes that need two sites have been categorised into different sub-types on the basis of how they cleave DNA with two or more copies of their site.^2^ The Type IIE enzymes, such as EcoRII, NaeI and Sau3AI,^27–29^ have two (or more)^30^ dissimilar DNA-binding clefts: one has the catalytic functions for cleaving the recognition sequence but is inactive unless a second copy of the sequence is bound to an activator cleft. The Type IIF enzymes, such as SfiI, NgoMIV and SgrAI, function as tetramers with two identical DNA-binding clefts, each made from two subunits^22,31^ but they are virtually inactive unless both clefts are filled with cognate DNA.^24,32^ Consequently, while both Type IIE and IIF enzymes cleave two-site substrates more rapidly than one-site substrates, Type IIE enzymes cut two-site DNA initially at just one site and then, in a much slower reaction, the second site.^33^ In contrast, Type IIF enzymes act concertedly, to give as their initial product DNA cut at both sites.^22,24,34,35^

Type II restriction enzymes have also been categorised on the basis of the positions at which they cleave the DNA relative to their recognition sequences.^2^ While many Type II enzymes cleave within palindromic sites, the Type IIS nucleases recognise asymmetric sequences and cleave both strands at specified positions on one side of the site.^3^ For example, FokI cleaves DNA downstream of the sequence GGATG, 9 and 13 bases away in “top” and “bottom” strands, respectively. Its recognition site is thus usually noted as GGATG(9/13). Most Type IIS nucleases, including FokI, have very low activities against DNA with one cognate site, and become active only after interacting with two sites.^36^ However, they vary in their modes of action at two DNA sites: some cut just one strand at one site,^13^ others both strands at one site, like Type IIE enzymes,^36–38^ while further examples cut both strands at both sites, like Type IIF enzymes.^39,40^

Categorisation of R-M systems from their positions of DNA cleavage yields a further subset,^2^ the Type IIB enzymes (Table 1) exemplified by BcgI.^41–45^ The Type IIB systems have bipartite recognition sequences that are usually asymmetric though some, such as BplI and FalI, have palindromic sites (Table 1).^3^ Their recognition sites look like sites for Type I rather than Type II systems but the Type IIB systems belong unambiguously to the II family because their endonucleases cut DNA at specified rather than random positions. Even so, the similarities between the Type I and the IIB systems extend to their functional organisations.^42,44,46–48^ Like Type I enzymes,^4^ each IIB system features a multi-functional protein with both methyltransferase and endonuclease activities. BcgI contains two polypeptides, A and B, in a 2:1 ratio.^42^ The A subunit has the amino acid sequences for both methylation and cleavage activities but by itself has neither activity. The B subunit resembles a DNA specificity subunit from a Type I enzyme and is thus likely to direct the protein to its recognition site.^44^ Several other Type IIB enzymes contain two subunits akin to the A and B subunits of BcgI,^49^ though in BplI these are at a 1:1 rather than a 2:1 ratio.^50^ In contrast, some IIB enzymes carry the endonuclease, the methyltransferase and the DNA recognition functions in three separate blocks of amino acid sequence within a single polypeptide.^46–48^ The IIB systems all require AdoMet for DNA methylation and Mg^2+^ for DNA cleavage but, as is common among combined restriction–modification proteins, many also need the methylation cofactor for their nuclease activity (Table 1).^43^

The positions of DNA cleavage by Type IIB nucleases are, like Type IIS enzymes, fixed distances from their recognition sites, 7–15 bases away depending on the system in question (Table 1).^3^ While the Type IIS enzymes make a double-strand break on one side of the site, the Type IIB nucleases make double-strand breaks on both sides of their sites,^41,46–50^ so cutting a total of four phosphodiester bonds at each site. They thus release their intact recognition sites from the remainder of the DNA on a short fragment, 30–35 bp long depending on the enzyme (Table 1). Perhaps as a result of remaining bound to its 34 bp product, the BcgI nuclease functions stoichiometrically, completing just one turnover, rather than catalysing multiple turnovers.^42–45^ However, the complete cleavage of a plasmid with two BcgI sites required less of the BcgI protein than a plasmid with one site, so it was suggested that BcgI interacts with two copies of its target sequence before cutting DNA.^45^ Many restriction enzymes have to bridge two sites before they can cleave DNA but it has yet to be determined whether this is a general characteristic of the Type IIB endonucleases, or unique to BcgI. This question was examined here by analysing the reactions of several Type IIB restriction proteins on DNA molecules that have either one or two copies of the relevant recognition sequence. The enzymes were from commercial suppliers, at concentrations given in terms of units (U) of activity rather than molarity, and were initially studied under the conditions advised by the supplier (Table 2).

## Results

### BcgI

Previous studies had shown that the amount of BcgI protein needed to cleave a plasmid with two BcgI sites (pBR322) was smaller than that required to cleave a plasmid with one site (pUC19), so it was suggested that BcgI needs to interact with two copies of its recognition site to cut DNA.^45^ However, the activities of restriction enzymes can be affected by non-specific sequences flanking the specific site and, for enzymes with bipartite recognition sequences as is the case with BcgI (Table 1), by the sequence of the spacer within the site.^17,51–53^ While one of the two BcgI sites in pBR322 is the same as that in pUC19, the other differs in both flanking and spacer sequences. The enhanced activity of BcgI on pBR322 could thus be due to the context of the novel site. Furthermore, the different amounts of enzyme needed to cleave these two plasmids may reflect different affinities for each DNA rather than different reaction rates. A derivative of pUC19 was therefore constructed that had two BcgI sites, both in the same sequence context as the pUC19 site: pDG5 (Supplementary Data, Figure S1).^18^

When tested at a concentration of BcgI that resulted in the cleavage of almost all of both one-site and two-site plasmids, the initial rate for the reaction on pUC19 was found to be about sixfold lower than that on pDG5 (Table 3). The difference in reaction rates cannot be due to either sequence differences surrounding the specified bp of the recognition sequence, nor to insufficient enzyme for complete cleavage of either DNA. Instead, the data support the previous proposal^45^ that BcgI interacts with two copies of its recognition site, preferentially *in cis*, before cutting the DNA.

Proteins can mediate long-range communications between distant DNA sites by either tracking along the 1-D contour of the DNA from one site to the other without losing contact with the DNA; or by binding concurrently to both sites upon the juxtaposition of the sites in 3-D space, so looping out the intervening DNA.^9,18^ These routes can be distinguished by comparing a DNA catenane containing two interlinked circles with one site in each ring to a single circle of DNA with two target sites.^33–35,54^ The catenane cannot support 1-D tracking from one site to the other^54^ but, since the 3-D distance between two sites in the separate rings of a supercoiled catenane will be similar to that between two sites in a supercoiled plasmid,^55^ the catenane should be as effective as the two-site plasmid for 3-D looping.^33^ This strategy was applied previously to several other restriction enzymes with the plasmid pMLE2,^34,35^ a DNA with two target sites for numerous Type II enzymes, including BcgI, interspersed with two directly repeated *res* sites from the transposon Tn*21*. It can be converted by Tn*21* resolvase into a catenane containing two interlinked rings of DNA with one BcgI site in each ring.

**Figure 1:**
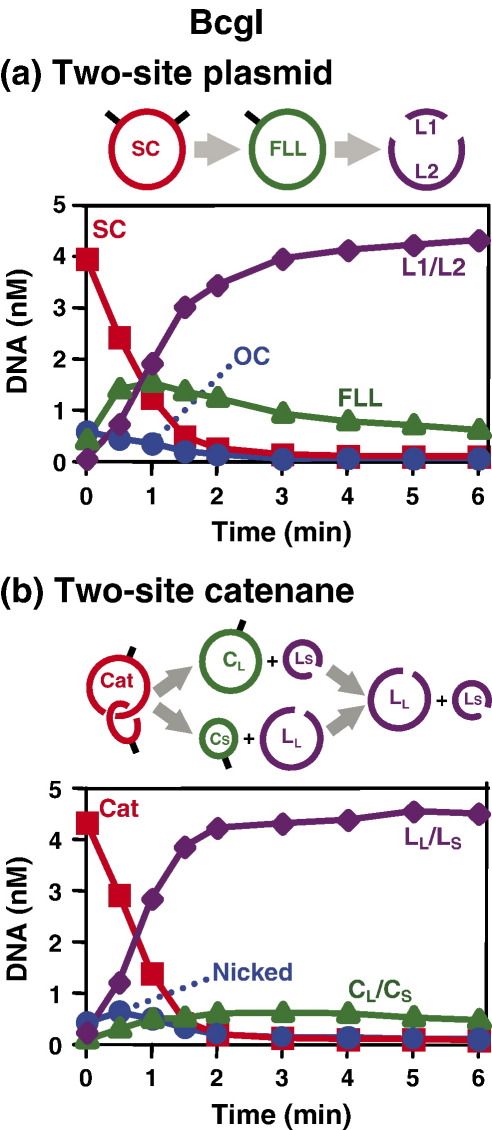
BcgI. The reactions contained 5 nM DNA (^3^H-labelled) and BcgI endonuclease (4 U) in 100 μl BcgI buffer at 37 °C. The DNA was: (a) pMLE2, a plasmid with two BcgI sites (hatch marks) and two *res* sites from Tn*21*; (b) the catenane created from pMLE2 by Tn*21* resolvase, with one BcgI site in each ring. Samples (6 μl) were taken from the reactions at the times indicated, mixed immediately with 4 μl of Stop-Mix and analysed by electrophoresis through agarose. The concentrations of the following forms of the DNA were measured: (a) the intact supercoiled form of the plasmid (SC, in red), the nicked form (OC, in blue), the full-length linear form cut in both strands at one BcgI site (FLL, in green) and the mean of the two linear products cut in both strands at both BcgI sites (L1/L2, in purple); (b) the intact catenane (Cat, in red), the sum of the catenanes nicked in either one or both rings (nicked, in blue), the mean of the two linear products from double-strand breaks in the individual rings (L_L_/L_S_, in purple), the mean of the two circular products left after cutting both strands in only the opposite ring (C_L_/C_S_, in green).

Initial rates for the reactions of BcgI on the parental plasmid with two BcgI sites (Figure 1(a)) and on the catenane with one BcgI site in each ring (Figure 1(b)) were measured from the decline in the concentration of the supercoiled substrates with time: the initial rates on the plasmid and on the catenane, 0.40 nM/min and 0.37 nM/min, respectively, were almost identical and in both cases considerably faster than that of 0.07 nM/min on a plasmid with one BcgI site under comparable conditions (data not shown). Moreover, almost all of the catenane was cleaved by BcgI in a highly concerted manner to give directly the final products with double-strand break(s) in both rings: neither the nicked forms, that would have arisen from single-strand breaks in one or both rings, nor the individual circles, from double-strand break(s) in the other ring, accumulated appreciably during the course of the reaction (Figure 1(b)). Hence, BcgI must bridge two copies of its recognition sequence through 3-D space and then introduce at least one double-strand break at each site before releasing the DNA.

### AloI, BaeI and BplI

To examine whether other Type IIB endonucleases need to interact with two sites, a series of plasmids were constructed with either one or two copies of their recognition sequences (Supplementary Data, Figures S1–S3). Most of the constructs involved cloning a duplex of synthetic oligonucleotides at one location in the vector, to give a plasmid with one target site for each enzyme: then inserting into that plasmid at a separate location a second copy of the duplex, to give a plasmid with two copies of each target. In these cases, the recognition sites for the test enzyme on the one-site substrate, and both of its sites on the two-site substrate, were all within the same sequence context. On the two-site substrates made here, the distances between the pairs of sites varied from 673 bp to 1288 bp (Table 3; on pDG5, the BcgI sites are 1810 bp apart). As the reactions were carried out on supercoiled plasmids, this variation is not significant. In a supercoiled plasmid, the mean separation of sites in 3-D space is only marginally affected by their 1-D contour separation along the DNA.^56^ Furthermore, even the shortest separations span several persistence lengths, thus nullifying any effect due to twisting and bending of the intervening DNA.^9^ A further consideration for the test plasmid is that proteins capable of bridging two asymmetric DNA sequences are often affected by the orientation of the sequences: some prefer directly repeated sites, others sites in inverted orientation.^40^ This variation was removed by using as the new two-site substrates only plasmids with inverted sites.

**Figure 2:**
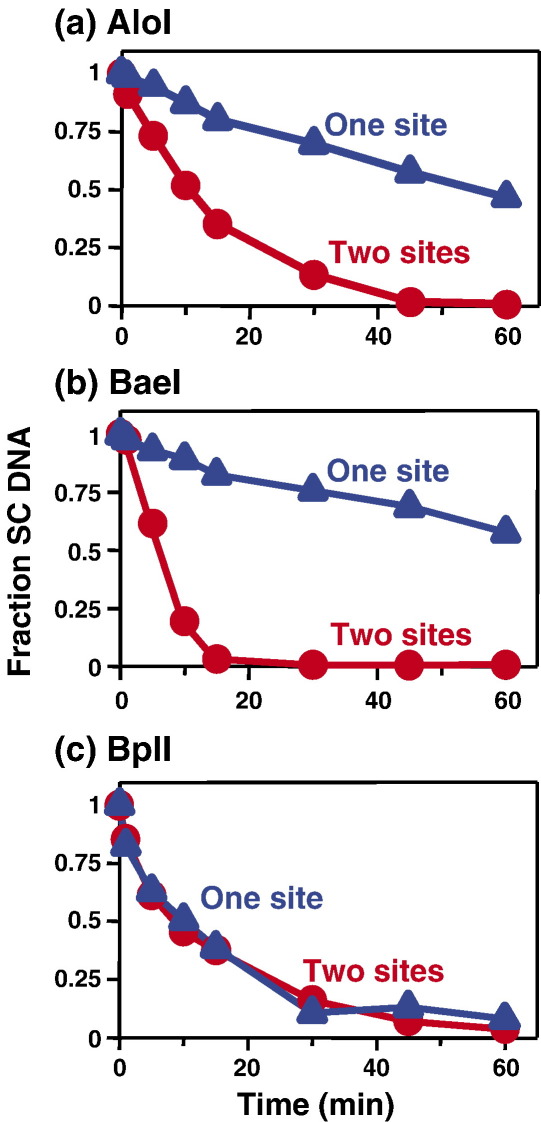
AloI, BaeI and BplI. Reactions contained, in 200 μl of the appropriate reaction buffer at the requisite temperature (Table 1), 5 nM ^3^H-labelled DNA and: (a) AloI endonuclease (1 U); (b) BaeI (1 U); (c) BplI (10 U). The DNA substrates were the SC forms of: pJM1 (blue triangles), a plasmid with one recognition site each for AloI, BaeI and BplI; pJM2 (red circles), a plasmid with two recognition sites each for AloI, BaeI and BplI (Supplementary Data, Figure S1). Samples (10 μl) were taken from the reactions at the times indicated and analysed as described in Materials and Methods. The concentrations of SC DNA still present at each time point are given as a fraction of that present at zero time.

Initial tests were carried out on three Type IIB enzymes, AloI, BaeI and BplI, as these embody several different aspects of the IIB systems (Table 1). Both BaeI and BplI contain two polypeptide chains, A and B, but these are present in BplI in a 1:1 rather than the 2:1 ratio seen with BcgI.^49,50^ Conversely, AloI is composed of a single polypeptide that carries endonuclease, methyltransferase and DNA recognition functions.^47^ BplI also differs from most Type IIB enzymes in having a palindromic recognition site while both BaeI and AloI have asymmetric targets, as is usual amongst IIB enzymes. Indeed, the recognition sequence for BaeI is the most asymmetric of the IIB sites; one segment of its bipartite sequence is 5 bp long, the other only 2 bp (Table 1). A plasmid with solitary recognition sites for AloI, BaeI and BplI, pJM1, was constructed and this used in turn to make a plasmid, pJM2, with two sites for each of these enzymes. Both AloI and BaeI cleaved the plasmid with two copies of their respective sites more rapidly than the plasmid with one copy (Figure 2(a) and (b)): fourfold faster in the case of AloI, similar to BcgI; by a larger factor, 13-fold, in the case of BaeI (Table 3). In contrast, BplI cleaved the supercoiled plasmids with one and two BplI sites at identical rates (Figure 2(c)).

**Figure 3:**
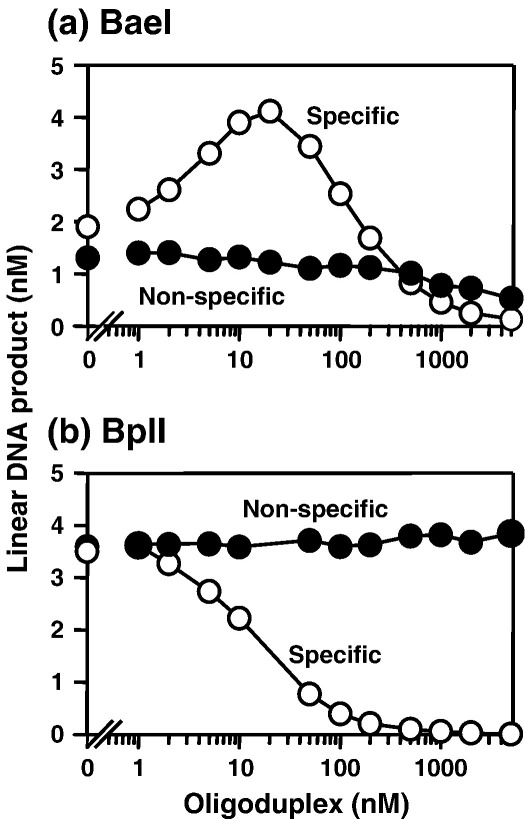
Tests for interactions *in trans* by BaeI and BplI. (a) Reactions in BaeI buffer (20 μl) contained BaeI endonuclease (0.1 U), 5 nM ^3^H-labelled pJM1 and the concentrations indicated on the *x*-axis of either a specific oligoduplex, duplex I (white circles), or a non-specific oligoduplex, duplex V (black circles). After 3 min at 25 °C, the reactions were quenched and the concentration of the linear DNA product determined. (b) Reactions were as for (a) except that the enzyme was BplI (1 U) and reactions were carried out at 37 °C in BplI buffer for 10 min. The plasmid pJM1 and duplex I both have one recognition site for BaeI and one for BplI (Supplementary Data, Figure S1). Duplex V has neither BaeI nor BplI sites (Supplementary Data, Figure S2).

Another strategy to determine whether a restriction enzyme needs two sites is to see if it can be activated to cleave a plasmid with one cognate site by an oligoduplex that has the recognition sequence.^20,27^ Interactions spanning separate molecules of supercoiled DNA are generally disfavoured so the interaction *in trans* needed to cleave the one-site plasmid can, at an appropriate concentration of duplex, be achieved more readily between plasmid and duplex than between two molecules of plasmid.^24,25^ However, higher concentrations of the duplex will inhibit plasmid cleavage as the enzyme becomes fully occupied with the duplex.^24,39^ When this test was applied to BaeI (Figure 3(a)), a specific oligoduplex with the cognate sequence for BaeI enhanced the cleavage of the plasmid with one BaeI site when present at relatively low concentrations, but abolished plasmid cleavage at higher concentrations. A control non-specific duplex lacking the sequence for BaeI failed to activate cleavage of the one-site plasmid, though high concentrations of this duplex led to a small reduction in plasmid cleavage, presumably due to the non-specific sequence acting as a weak inhibitor. Similar results were obtained when specific and non-specific duplexes were added to AloI reactions (data not shown). With BplI (Figure 3(b)), the non-specific duplex again had no effect on the cleavage of the one-site plasmid but in this case the specific duplex with the BplI site acted solely as an inhibitor of plasmid cleavage: no activation was observed at any duplex concentration tested.

Taken together, the above data show that both AloI and BaeI need to interact with two copies of their recognition sites in order to cut DNA. The faster reaction rates of these enzymes on the two-site plasmid, relative to the one-site DNA, are likely due to the intrinsic preference for interactions *in cis*, spanning sites in the same molecule of DNA, over interactions *in trans*, across separate molecules. Nevertheless the reactions of both AloI and BaeI on the one-site plasmid could be activated by adding a duplex with the requisite recognition sequence. These two enzymes are therefore not limited to sites *in cis*, but can also become active by binding two sites *in trans*. In contrast, the reactions of BplI on one and two-site plasmids, and the effect of a specific duplex on its one-site reaction, all match the expectations of a restriction enzyme that acts at individual sites, like EcoRV or BglI.^17–19^

### Other Type IIB enzymes

The above experiments raise two possibilities. One is that the requirement of two recognition sites, as seen with BcgI, BaeI and AloI, is a general property of the Type IIB enzymes and that BplI is a solitary exception. The other is that the requirement for two sites is shared by only a fraction of the Type IIB enzymes and that BplI represents a major portion of these enzymes. Further plasmids with one or two copies of the requisite recognition sites were constructed to test most of the other Type IIB enzymes currently available (Table 1): AlfI, BsaXI, CspCI, FalI, PpiI and PsrI. When tested under the recommended reaction conditions for the enzyme in question (Table 2), four out of the six additional enzymes, AlfI, CspI, FalI and PpiI, cleaved their two-site substrate at significantly faster rates than their one-site substrate: by factors that varied from sixfold with AlfI and FalI to 43-fold with CspCI (Table 3).

AlfI and FalI recognise palindromic sequences, a feature of the BplI system but not of many other Type IIB systems (Table 1). Hence, Type IIB enzymes with palindromic sites do not necessarily act like BplI. The reactions of FalI on the plasmid with one FalI site were also examined in the presence of varied concentrations of oligoduplexes with and without its recognition sequence (data not shown). The specific oligoduplex activated FalI-cleavage of its one-site substrate, exactly like BaeI (Figure 3(a)), while the non-specific DNA had no effect apart from a slight inhibition at high concentrations, again like BaeI. FalI is therefore a Type IIB enzyme with a palindromic recognition sequence but which has to interact with two copies of its sequence to cut DNA, as is also AlfI (see below; Figure 5(a)).

**Figure 4:**
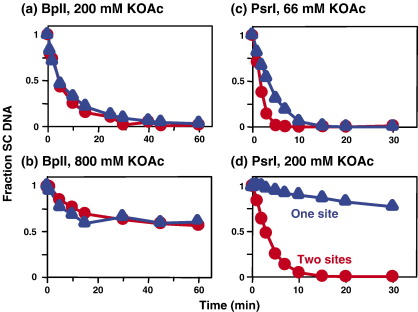
Varied ionic strengths with BplI and PsrI. For (a) and (b), the reactions contained, in 200 μl of buffer at 37 °C, BplI endonuclease (10 U) and 5 nM SC DNA (^3^H-labeled): either pJM1 (which has one BplI site; blue triangles) or pJM2 (with two BplI sites; red circles). The buffer was the same as BplI buffer (Table 2) except that the concentration of KOAc had been changed from 66 mM to either (a) 200 mM or (b) 800 mM. For (c) and (d), reactions at 30 °C contained, in 200 μl of buffer, PsrI (2 U) and ^3^H-labelled SC plasmid (5 nM): either pJM3 (one PsrI site; blue triangles) or pJM4 (two PsrI sites; red circles). The buffer was either PsrI buffer (c) or the same buffer but with 200 mM KOAc instead of 66 mM (d). Samples taken from the reactions at the times indicated were analysed as described in Materials and Methods to determine the concentration of the SC substrate left at each time point: the concentration is shown as a fraction of the SC DNA present at zero time.

Under their standard reaction conditions, the remaining two enzymes, BsaXI and PsrI, cleaved their two-site plasmids at rates that were ≤2-fold faster than those on their one-site substrates (Table 3; Figure 4(c)). A restriction enzyme that acts at individual sites will cleave a two-site DNA twice as fast as a one-site DNA if the DNA concentration is above that of the enzyme but below the *K*_m_ value, simply due to the twofold difference in substrate concentration (i.e. recognition sites). Alternatively, if present at a >2-fold excess over the DNA, an enzyme that acts at individual sites will again carry out its initial reaction on a two-site DNA twice as fast as on a one-site DNA, as two molecules of the enzyme can then act simultaneously on the two-site DNA, one at each site. However, Type II restriction enzymes that need two sites often cleave their one and two-site substrates at similar rates in reactions at low ionic strength, but cleave the two-site DNA faster than the one-site DNA at high ionic strength.^16,18,26,39^ Consequently, to find out why BsaXI and PsrI cleave their one and two-site substrates at similar rates, and why BplI cleaves its equivalent substrates at identical rates, the reactions of these three enzymes were studied in buffers of varied ionic strength (Figure 4; Table 3).

The recommended reaction buffer for BplI contains 66 mM KOAc (Table 2) and, under these conditions, BplI cleaved to completion its one and its two-site substrates at identical rates (Figure 2(c)). An increase in the concentration of KOAc to 200 mM again allowed complete cleavage of both substrates, even more rapidly than at 66 mM, but a further increase to 800 mM led to incomplete cleavage of both substrates (Figure 4(a) and (b)). However, at all KOAc concentrations tested, the progress curves for the BplI reactions on its one and two-site substrates were superimposable. Similar results were obtained when the ionic strength was varied by adding NaCl instead of KOAc: the addition of NaCl affected both the rates and amplitudes of the BplI reactions, but NaCl had the same effect on the one-site and the two-site reactions (data not shown). In contrast, while PsrI had cleaved its two-site DNA at a marginally higher rate than its one-site DNA in a reaction buffer with 66 mM KOAc (Figure 4(c)), it cleaved the two-site DNA at a considerably faster rate than the one-site DNA in the presence of 200 mM KOAc (Figure 4(d)): 12-fold faster instead of the twofold difference under standard conditions (Table 3). For BsaXI, the ratio of its reaction rates on its two and one-site substrates likewise increased as the KOAc concentration was raised: from 1.6-fold at 50 mM to fourfold at 200 mM (Table 3).

### Modes of action

**Figure 5:**
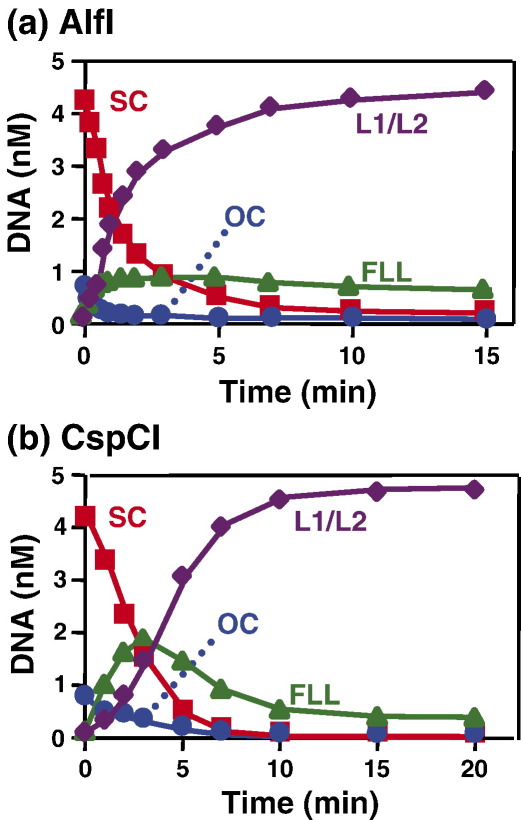
Modes of action by AlfI and CspCI. The reaction in (a) contained, in 200 μl AloI buffer with 50 μM AdoMet (Table 2) at 20 °C, AlfI endonuclease (2 U) and 5 nM pIS2 (a plasmid with two AlfI sites). The reaction in (b) contained, in 200 μl of BaeI buffer (Table 2) at 37 °C, CspCI (2 U) and 5 nM pIS1 (a plasmid with two CspCI sites). In both reactions, the substrate was ^3^H-labelled and ∼85% was initially in its supercoiled state. Samples were taken from the reactions at the times indicated and analysed as described in Materials and Methods to determine concentrations of the following forms of the DNA: the supercoiled plasmid (marked SC, in red), the nicked form (OC, in blue), the full-length linear form with at least one double-strand break at one recognition site (FLL, in green) and the mean of the two linear products with double-strand breaks at both sites (L1/L2, in purple).

Apart from BplI, all of the Type IIB enzymes tested here are capable of cleaving plasmids with two cognate sites more rapidly than plasmids with a single site, so they must act on the two-site DNA by bridging sites *in cis*. Among the restriction enzymes that bridge DNA sites *in cis*, the Type IIE systems first cleave one site and then in a separate reaction the other, often at a much slower rate.^20,21^ In contrast, the Type IIF enzymes act concertedly on two-site substrates and liberate as their initial product the DNA cut in both sites, bypassing intermediates cut at one site.^19,22,24^ These mechanisms can be distinguished by examining the transient intermediates formed during their reactions on a supercoiled (SC) DNA with two recognition sites.^9,25,33^ Such a DNA can be cleaved to give, successively: the open-circle (OC) form cut in one strand at one or both sites; the full-length linear (FLL) form cut in both strands at one site; and, as the end products, the two linear fragments after cutting both sites. If a restriction enzyme cleaves the DNA by means of separate but kinetically equal reactions at each site, the amount of the FLL form should rise to a maximum of 40% of the total DNA before declining, upon cleavage of the second site.^17,57^ If it cleaves one site more rapidly than the other, as is the case with Type IIE enzymes,^30,33^ the amount of the FLL form will reach a maximum of >40% of the total. Conversely, if the enzyme first cuts one site at a slow rate and then the second site at a faster rate, as is the case with the Type IIF enzymes,^16,24^ the maximal amount of FLL DNA will be <40% of the total. Three examples of this strategy are shown: AlfI (Figure 5(a)), CspCI (Figure 5(b)) and BcgI (Figure 1(a)).

In all three examples, and also with all of the other Type IIB enzymes examined here apart from AloI (data not shown), their reactions yielded none of the OC form of the DNA that would have arisen if products cut in one strand, at one or both sites, had accumulated during the reaction. A SC DNA with two recognition sites for a Type IIB enzyme has eight scissile phosphodiester bonds, in top and bottom strands on either side of both sites. Cleavage of any one of these eight bonds will convert the SC to the OC form but if the next phosphodiester bond to be cleaved is chosen at random from the seven remaining targets, six out of the seven leave the DNA in its OC state and only one, that opposite the initial nick, will result in the OC form being converted to the linear product with one double-strand break. Hence, Type IIB enzymes might be expected to generate large amounts of the OC intermediate before creating any linear product. The observation that no OC DNA accumulated shows instead that, after cutting one strand at a single locus, Type IIB enzymes must cut the second strand at that locus very shortly thereafter, within the lifetime of a single DNA–enzyme complex.

The reaction of the AlfI endonuclease on its two-site substrate yielded not only, as noted above, none of the OC form but also very little of the FLL form (Figure 5(a)). (No distinction is made here between the products from bilateral as opposed to unilateral cleavages at individual sites: see Materials and Methods.) The amount of FLL DNA reached a maximum at about 15% of the total, far below the value of 40% expected for kinetically equal reactions at each site. The DNA cut at one AlfI site is therefore almost always cleaved at the other site before being liberated. Hence, after bridging two copies of its recognition sequence, AlfI acts concurrently at both sites in a highly concerted manner. The reaction profile of AlfI is thus reminiscent of SfiI and the other Type IIF restriction enzymes.^16,24,33^ Several other Type IIB enzymes also converted major fractions of their two-site substrates directly to the final products with double-strand breaks at both sites, liberating none of the OC and only small amounts of the FLL intermediate: they included FalI, PpiI and PsrI (data not shown). AlfI and the other Type IIB enzymes that act in this manner therefore fall into the Type IIF category.

On the other hand, the CspCI endonuclease converted the SC substrate with two CspCI sites first to the FLL form and only later, after a lag phase, to the two linear products cut at both sites: as before, intermediates cut in one strand were not detected (Figure 5(b)). Hence, CspCI first cuts both DNA strands at one recognition site, and then in a separate reaction, creates one or more double-strand breaks at the other site. The amount of FLL DNA formed during the CspCI reaction reached a maximum of ∼40% of the total DNA. This level matches the expectation for a restriction enzyme that cleaves a two-site substrate in separate but equal reactions at each site.^17,57^ AloI, BaeI and BsaXI also cleaved their two-site substrates to give first the FLL linear form, again to a maximal yield of ∼40% of the total, and only later the linear fragments with double-strand breaks at both sites. Though these enzymes all need to bind two copies of their recognition sites to become fully active, they cleave each site in separate reactions.

In the reaction of BcgI on pMLE2, a plasmid with two BcgI sites (Figure 1(a)), the FLL form of the DNA was observed as a transient intermediate but even at its peak, the FLL species constituted <30% of the DNA, below the level indicative of two separate but equal reactions. This shows that BcgI sometimes dissociates from this plasmid after cutting one site but sometimes cuts both sites before dissociating. Other plasmids that have two BcgI sites, but with different flanking/spacer sequences from those on pMLE2, are cleaved without releasing any of the FLL DNA (S. Ganguly, J.J.T.M. & D.M.G., unpublished results). Moreover, BcgI cleaved the catenane derived from pMLE2 in a highly concerted manner: it released virtually none of the individual circles that would have been liberated by making a double-strand break in one ring (Figure 1(b)). Hence, BcgI rarely dissociates from the catenane after cutting just one site and instead remains bound until it has opened both rings. Other restriction enzymes were observed previously to act more concertedly on catenanes with one target site in each ring than on plasmids with two sites *in cis*.^33^ A synaptic complex between two sites in the separate rings of a catenane is likely to be more stable than one spanning sites *in cis,* as the juxtaposition of the sites in the catenane will not impose any deformation of the intervening DNA. The enzyme may therefore be able to cut both sites within the lifetime of the catenane synapse but not within the lifetime of the plasmid synapse.

Amongst the Type IIB enzymes that cleave plasmids with two cognate sites more rapidly than plasmids with one target, the largest differences observed under standard reaction conditions were with BaeI and CspCI, which cleaved their two-site substrates 13 and 43-fold faster, respectively, than their one-site substrates (Table 3). Yet these two enzymes cleaved two-site plasmids by means of separate but kinetically equal reactions at each site (Figure 5(a)). To determine how these two enzymes communicate between distant DNA sites, their reactions were studied on catenane substrates that had in each ring either one BaeI site or one CspCI site (data not shown). When tested with the relevant enzyme, the catenanes were cleaved at similar rates to the parental two-site plasmid for that enzyme, in both cases more rapidly than the corresponding one-site plasmid. Moreover, like BcgI (Figure 1(b)), both BaeI and CspCI cleaved the catenanes much more concertedly than the plasmids: they released almost none of the individual circles from cutting the catenane in a single ring. Hence, both BaeI and CspCI act rapidly on DNA with two copies of their recognition sites by first spanning the sites through 3-D space, as opposed to tracking along the DNA. They can then act concertedly at both sites. BaeI and CspCI thus also belong in the Type IIF category of restriction endonucleases.

## Discussion

### The general case

The Type IIB endonucleases constitute a discrete subset of the Type II R/M systems,^2^ delineated by their bipartite recognition sequences and by their bilateral sites of DNA cleavage either side of their recognition sites (Table 1). To determine whether Type IIB enzymes need to interact with two target sites in order to cleave DNA, the reactions of ten IIB nucleases were examined on plasmids that carry either one or two copies of the requisite sequence. With one exception, BplI, they all cleaved their two-site substrates more rapidly than the one-site substrates (Table 3) though two, BsaXI and PpiI, gave only twofold (or smaller) differences under their standard reaction conditions. These two both exhibited larger differences at elevated ionic strengths (Figure 4, Table 3). Moreover, all of the Type IIB enzymes tested against catenanes cleaved the catenane with one site in each ring as readily as the parental plasmid with two sites in a single ring (Figure 1). In addition, in all cases tested apart from BplI, the Type IIB enzymes cleaved plasmids with a single cognate site at enhanced rates in the presence of cognate oligoduplexes (Figure 3).

These experiments confirm the original proposal of Kong & Smith,^45^ that BcgI interacts with two copies of its recognition sequence before cutting DNA, and show further that the vast majority of the Type IIB enzymes act in the same way. Proteins that interact with distant DNA sites intrinsically prefer sites *in cis* over sites *in trans*,^9,24,25^ which accounts for why these enzymes cleave the two-site plasmids more rapidly than their one-site substrates. Nevertheless, the catenane experiments show that they capture their two sites *in cis* through 3-D space and not by tracking along the DNA, like the Type I enzymes.^34,54^ They can, however, also act *in trans*, by forming a complex bridging one recognition site on a plasmid and another on an oligoduplex.

The Type IIB endonucleases that cleave DNA after binding two cognate sites include some composed of two polypeptide chains, such as BcgI and BaeI,^42,49^ and some composed of a single polypeptide, such as AloI and PpiI.^46–48^ They also include some with asymmetric recognition sequences, like BsaXI, CspCI and PsrI, and some with palindromic sites, like AlfI and FalI. Furthermore, they include some that require AdoMet for their nuclease activities, *viz*. BcgI and CspCI, and others with no requirement for AdoMet, *viz*. BsaXI and PsrI (Table 1). The need to interact with two sites before cutting the DNA is thus a general property of the Type IIB enzymes, common to a wide range of these enzymes even though they differ from each other in many other respects. Two main mechanisms exist for the Type II restriction enzymes that use two sites:^9,19–21,33^ some, the Type IIE systems, employ one site as an activator to enhance cleavage of another site;^27–30^ others, the Type IIF systems, act concertedly at both sites.^22,24,32–35^ The Type IIB nucleases fall into the latter category.

This study extends the range of restriction endonucleases that are currently known to cleave DNA only after interacting with two target sites. In addition to the Type I, III and IV systems,^1,4,5^ which constitute ∼60% of the R-M systems found in nature,^3^ the set includes a substantial fraction of the Type II enzymes: a portion of those that act within palindromic sites;^18^ the majority of the Type IIS systems that act on one side of an asymmetric site;^36–40^ and now, from this study, essentially all of the Type IIB enzymes that act bilaterally either side of a bipartite sequence. The enzymes that need two sites may therefore constitute 80% of the total and those that act at individual sites only 20% of the restriction enzymes present *in vivo*, even though the latter include the so-called archetypes like EcoRI, EcoRV and BamHI, the enzymes most widely used as tools for DNA manipulations *in vitro*.^58,59^ Though enzymes acting at individual sites might seem to be more efficient at restricting DNA than those requiring two sites, the widespread requirement for two sites indicates an advantage over the one-site systems.^19^ The need to interact with two copies of the recognition sequence before cleaving the DNA, rather than a single copy, may function as a double-check to ensure these enzymes only cleave DNA in response to the cognate sequence, and thus avoid untoward reactions at non-cognate sites.^9,38,60^ However, other restriction enzymes can utilise a cognate site to initiate cleavage of a non-cognate site.^22,35,61,62^ In these cases, the bridging interaction between cognate and non-cognate sites serves to broaden the range of sequences that the enzyme cleaves.^61^

### The special case

On the other hand, one of the ten Type IIB enzymes tested, BplI, showed none of the characteristics of a restriction enzyme that interacts with two sites. Instead, BplI catalyses separate and independent reactions at each copy of its recognition site, in the same manner as the standard restriction enzymes like EcoRV.^19^ Under all reaction conditions employed with BplI, the progress curves for the utilisation of its one-site and two-site substrates were superimposable, even though its overall activity varied considerably over the range of conditions tested (Figures 2(c), 4(a) and (b)). Unlike Psr I for example, which cleaved its one and two-site substrates at similar rates at low ionic strength but at dissimilar rates at higher strengths (Figure 4(c) and (d)), no difference was observed with BplI even when the salt concentration was too high to allow complete DNA cleavage. Moreover, BplI cleaved the plasmid with two BplI sites by means of separate reactions at each site, albeit at the same rate (data not shown). In addition, an oligoduplex with the recognition sequence for BplI inhibited rather than enhanced its cleavage of a plasmid with one BplI site (Figure 3(b)), which again indicates that BplI can bind only one copy of its recognition sequence at a time.

BplI clearly acts in an atypical manner for a Type IIB endonuclease, but its unusual properties cannot be due to its palindromic recognition sequence. Though most Type IIB enzymes recognise asymmetric sites (Table 1), some such as AlfI and FalI have palindromic sites yet still need two sites to cleave DNA. However, the unique properties of BplI might be a consequence of its subunit composition. Like BcgI, BplI is made up of two polypeptide chains but seemingly in a 1:1 ratio instead of the 2:1 ratio reported for BcgI.^42,50^ A related possibility is that BplI is not really a Type IIB enzyme, and is instead a novel Type IIS system with a palindromic recognition site. The restriction enzymes currently considered as Type IIS systems all have asymmetric sites and cut DNA at specified positions on one side of the site.^2,3^ A protein must bind to an asymmetric sequence in one particular orientation, which fixes the position of its catalytic residues on the DNA,^57^ but it can bind to a palindromic site in either of two orientations. BplI cleaves DNA either side of its site 8 and 13 bases away (Table 1)^50^ so on 50% of the occasions that it binds to its site, its catalytic functions may be located 8/13 bases to the “left” of the site and, on the 50% of the time, 13/8 bases to the “right” of the site. A Type IIS enzyme with a palindromic site would thus make bilateral cleavages either side of its site in the characteristic manner of a Type IIB enzyme. Though most of the Type IIS enzymes need to interact with two sites for full activity,^13,37–40^ some need only one site.^36^

## Materials and Methods

### Enzymes

Tn*21* resolvase was purified as before.^33,34,54^ All other enzymes were obtained from commercial suppliers, stored at –20 °C and used essentially as advised by the supplier. The suppliers of the Type IIB restriction enzymes studied here, and the primary reaction buffers for each enzyme, are listed in Table 2. The concentrations of these enzymes were given in terms of units (U) of enzyme activity per ml, as specified by the supplier. While all of the commercial enzymes were free from non-specific nucleases, the purities of the preparations were not known and could not be assessed here. Most of the supplied samples contained insufficient amounts of protein for further analysis: the number of units in each purchase varied from 50 U to 1250 U and Type II restriction enzymes typically have specific activities of 10^6^–10^7^ U/mg of protein.^10,19^

### DNA

The plasmid pUC19^63^ and its derivatives pMDS2(a),^34^ pMLE1 and pMLE2,^33^ and pDG5,^18^ have been described. Specifically, pDG5 had been constructed from pMLE1 by inserting a 56 bp duplex that contained the same sequence as that around the BcgI site in pUC19: pDG5 is thus a 3806 bp plasmid with two BcgI sites 1810 bp apart that are identical to each other and to that in pUC19.

Further plasmids, with one or two copies of Type IIB recognition sites were constructed from pDG5 and pMDS2a (Supplementary Data, Figures S1–S3). Typically, the vector was first linearised with a restriction enzyme and then ligated to a DNA duplex with appropriate single-strand extensions: the duplexes carried recognition sequences for several Type IIB enzymes and were made by annealing pairs of synthetic oligodeoxyribonucleotides (MWG Biotech). The ligation mixtures were used to transform *Escherichia coli* HB101 and the plasmids from several transformants sequenced across the site of the insertion (University of Dundee Sequencing Service). From each ligation, some transformants were found to contain a plasmid with a single copy of the insert at the requisite site. These plasmids were then used as vectors to clone a second duplex at a separate site, to give plasmids with two inserts in either inverted or directly repeated orientation. The constructs with inverted (head-to-head) inserts were used as the two-site substrates.

The transformants were cultured in M9 minimal media containing 37 MBq/l [*methyl*-^3^H]thymidine (GE Healthcare) and the plasmids purified by CsCl density-gradient centrifugations.^31–34^ The preparations generally contained 80–95% supercoiled monomeric plasmid, with 5–20% open-circle and dimeric forms. The supercoiled forms of pMLE2, pJM2 and pIS2 were converted into catenanes by reactions with Tn*21* resolvase at a 24-fold molar excess over the plasmid, as noted before.^18,33,54^ DNA concentrations were evaluated from *A*_260_ measurements.

### Reactions

Reactions were usually carried out by adding 0.5–4 μl of the restriction enzyme (diluted, if necessary, in the buffer advised by the supplier) to 200 μl of ^3^H-labelled DNA (5 nM) in the appropriate buffer at the requisite temperature (Table 2). One 10 μl aliquot (the zero-time point control) was removed before adding the enzyme and further aliquots at timed intervals thereafter. The aliquots were mixed immediately with 5 μl of an EDTA Stop-Mix.^24,32,38^ In some instances, reactions were carried out in 20 μl volumes containing constant amounts of enzyme and plasmid but with varied concentrations of an oligoduplex: some duplexes contained, others lacked, the recognition sequence for the test enzyme. After a fixed time interval, these reactions were quenched with Stop-Mix as described above.

The quenched samples were analysed by electrophoresis through agarose.^26,40^ From reactions on plasmid substrates, the following forms of the DNA were separated: the intact supercoiled (SC) plasmid; the nicked open-circle (OC) form; the full-length linear (FLL) DNA cut in both strands at one site; and, for substrates with two sites, the two linear products (L1 and L2) from making double-strand breaks at both sites.^16,25^ With catenane substrates, the intact catenane was separated from the catenanes with nicks in one or both rings; and from both the separate rings and the linear products due to double-strand breaks in one or both sites.^7,33–35^ Type IIB enzymes cleave DNA on both sides of their recognition sites, but the linear product from cutting on one side of a site is only 27– 33 bp longer than that cleaved on both sides of the same site. As the plasmids used here were ∼4 kb in size, with ∼1 kb between the sites in the two-site substrates, the products from bilateral cleavages were not separated from those with unilateral cleavages. Moreover, the 27–33 bp fragments excised from the remainder of the DNA were too small to detect on the agarose gels used here. It was assumed that DNA products cut in both strands at one or both recognition sites had been cleaved bilaterally at those sites.

Segments of the gels were analysed by scintillation counting,^25,40,57^ to determine the concentration of each form at each time point. The concentrations shown in Figures 1–5 are the means from three independent experiments: for clarity, the error bars for the standard deviations (typically <10% of the mean values) have been omitted. Initial rates were evaluated from the mean values for the concentration of supercoiled DNA, by using GRAFIT (Erithacus Software, Horley, UK) to fit to a linear slope the decrease in these concentrations with time, starting at time zero and extending over the zero-order stage of the reaction: the errors in the reaction velocities cited in Table 3 are the standard errors from the fitting procedure.^17,57^ The errors on the ratios of the rates on the one and two-site plasmids were calculated from the error limits on each of the individual rates.

For the majority of the Type IIB enzymes tested here, the concentrations of their substrate ([S]) declined linearly during their reactions, for ≥50% of the total reaction: for example, BaeI (Figure 2(b)). However, for some such as AloI (Figure 2(a)), [S] declined in an exponential rather than a linear fashion. For substrate utilization to follow an exponential curve, one of the following must pertain: the reaction contains [E_0_] > [S] and thus has the characteristic first-order kinetics for a single-turnover reaction; or it is under multiple-turnover (steady-state) conditions with [E_0_] < [S], but with an initial [S] below the *K*_m_ value so that the reaction velocity then declines in parallel with [S].^52^ The former may apply to BcgI, as this enzyme seems to act stoichiometrically rather than catalytically.^42,45^ Further experiments using BcgI that had been purified in this laboratory (S. Ganguly, S. Milsom, J.J.T.M & S.E.H., unpublished results) show that all of the BcgI reactions described here contained enzyme in excess of the substrate. It has yet to be determined which of these two possibilities apply to the other Type IIB enzymes that give exponential curves, as their concentrations were known only in terms of enzyme units, not molarities. On the other hand, a linear decline in [S] can only arise from a zero-order steady-state reaction involving multiple turnovers of the enzyme. Consequently, to permit comparisons of different enzymes, all of the reaction rates were evaluated as zero-order velocities from the decline in [S] with time over the initial portion of the reaction.

## Figures and Tables

**Table 1:** Type IIB restriction–modification systems

Enzyme	Source organism	Recognition sequence^a^	Excised fragment^b^ (bp)	No. peptides^c^ (and proposed ratio)	Nuclease requirement for AdoMet	Reference
AlfI	*Acinetobacter lwoffi* BH 32	(10/12) GCA-N_6_-TGC (12/10)	34	–	Yes	^3^
AloI	*Acinetobacter lwoffi* Ks 4–8	(7/12) GAAC-N_6_-TCC (12/7)^V^	32	1	No	^47^
BaeI	*Bacillus sphaericus*	(10/15) AC-N_4_-GTAYC (12/7)^V^	33	2 (−)	Yes	^49^
BcgI	*Bacillus coagulans*	(10/12) CGA-N_6_-TGC (12/10)	34	2 (A_2_B_1_)	Yes	^41–43^
BplI	*Bacillus pumilus*	(8/13) GAG-N_5_-CTC (13/8)	32	2 (A_1_B_1_)	Yes	^50^
BsaXI	*Bacillus stearothermophilus*	(9/12) AC-N_5_-CTCC (10/7)	30	–	No	^3^
CspCI	*Citrobacter* species 2144	(11/13) CAA-N_5_-GTGG (12/10)^V^	35	–	Yes	^3^
FalI	*Flavobacterium aquatile*	(8/13) AAG-N_5_-CTT (13/8)	32	–	Yes	^3^
PpiI	*Pseudomonas putida*	(7/12) GAAC-N_5_-CTC (13/8)^V^	32	1	No	^48^
PsrI	*Pseudomonas stutzeri*	(7/12) GAAC-N_6_-TAC (12/7)	32	–	No	^3^

All of the Type IIB restriction endonucleases analysed here are listed, with the bacterial species from which they were originally isolated.

a In the recognition sequences, the pairs of numbers either side of the nucleotide sequence shown indicate the number of bases between the specified sequence and the point of cleavage, firstly in the strand shown, secondly in the complementary strand. N denotes any base and Y a pyrimidine.

b The length of the DNA fragment that the nuclease excises from its substrate is given as the number of bases in each strand.

c Whenever known (– indicates not known), the number of different polypeptide chains is as shown: for two-polypeptide systems, the ratio is given.

V Indicates that these particular enzymes can cleave at varied positions that differ from the canonical by 1 or 2 bp.

**Table 2:** Enzyme suppliers and reaction conditions

Enzyme	Supplier	Standard reaction buffer^a^
AlfI	Fermentas, Lithuania	AloI buffer with 50 μM AdoMet at 20 °C
AloI	Fermentas	10 mM Tris–HCl, 100 mM KCl, 10 mM MgCl_2_ (pH 8.5) at 30 °C
BaeI	New England Biolabs, USA	10 mM Tris–HCl, 50 mM NaCl, 10 mM MgCl_2_, 1 mM DTT, 50 μM AdoMet (pH 7.9) at 25 °C
BcgI	New England Biolabs	10 mM Tris–HCl, 100 mM NaCl, 10 mM MgCl_2_, 1 mM DTT, 20 μM AdoMet (pH 8.5) at 37 °C
BplI	Fermentas	33 mM Tris–OAc, 66 mM KOAc, 10 mM Mg(OAc)_2_, 50 μM AdoMet (pH 7.9) at 37 °C
BsaXI	New England Biolabs	20 mM Tris–OAc, 50 mM KOAc, 10 mM Mg(OAc)_2_, 1 mM DTT (pH 7.9) at 37 °C
CspCI	New England Biolabs	BaeI buffer at 37 °C
FalI	SibEnzyme, Russia	BcgI buffer with 50 μM AdoMet at 37 °C.
PpiI	Fermentas	AloI buffer at 30 °C
PsrI	SibEnzyme	33 mM Tris–OAc, 66 mM KOAc, 10 mM Mg(OAc)_2_, 1 mM DTT (pH 7.9) at 30 °C

a All reaction buffers also contained 0.1 mg/ml of bovine serum albumin.

**Table 3:** Reactions of Type IIB restriction enzymes on plasmids with one or two recognition sites

Enzyme	Reaction conditions^a^	One-site substrate^b^	Two-site substrate^b^ (site separation)^c^	Initial rates (nM/min) on:^d^		Ratio: 2-site rate/1-site rate
				One-site DNA	Two-site DNA	
BcgI	40 U/ml in BcgI buffer	pUC19	pDG5 (1810 bp)	0.052 ± 0.004	0.35 ± 0.03	6.7 ± 1.1
AloI	5 U/ml in AloI buffer	pJM1	pJM2 (682 bp)	0.053 ± 0.002	0.20 ± 0.01	3.8 ± 0.3
BaeI	5 U/ml in BaeI buffer	pJM1	pJM2 (703 bp)	0.027 ± 0.004	0.35 ± 0.01	13.0 ± 2.3
BplI	50 U/ml in BplI buffer	pJM1	pJM2 (694 bp)	0.27 ± 0.08	0.30 ± 0.05	1.1 ± 0.6
AlfI	10 U /ml in AlfI buffer	pIS1	pIS2 (1072 bp)	0.31 ± 0.01	1.80 ± 0.15	5.8 ± 0.6
CspCI	10 U/ml in BaeI buffer	pMDS2(a)	pIS1 (1288 bp)	0.021 ± 0.006	0.90 ± 0.03	42.9 ± 14.8
FalI	15 U/ml in BcgI buffer	pJM3	pJM4 (673 bp)	0.12 ± 0.02	0.75 ± 0.08	6.3 ± 1.7.
PpiI	20 U/ml in AloI buffer	pJLV2	pDG5 (842 bp)	0.18 ± 0.01	1.59 ± 0.08	8.8 ± 0.8
BsaXI	5 U/ml in BsaXI buffer (50 mM KOAc)	pDG5	pKW1 (875 bp)	0.13 ± 0.01	0.21 ± 0.02	1.6 ± 0.3
PsrI	10 U/ml in PsrI buffer (66 mM KOAc)	pJM3	pJM4 (681 bp)	0.58 ± 0.03	1.18 ± 0.08	2.0 ± 0.2
BsaXI	5 U/ml in BsaXI buffer with 200 mM KOAc	pDG5	pKW1 (875 bp)	0.025 ± 0.003	0.10 ± 0.01	4.0 ± 0.9
PsrI	10 U/ml in PsrI buffer with 200 mM KOAc	pJM3	pJM4 (681 bp)	0.050 ± 0.005	0.62 ± 0.04	12.4 ± 2.1

a Buffer compositions are as in Table 2.

b Plasmid constructs are given in Supplementary Data, Figures S1–S3.

c Site separations correspond to the shorter of the two centre-to-centre distances in the circular plasmid.

d Three or more repeats of each reaction were carried out and a mean value calculated for the concentration of supercoiled DNA substrate at each individual time point. The mean values were fitted to a linear slope to give the initial rates shown. Errors on the rates indicate standard errors from the fitting procedure. Compound errors are recorded for the ratios of the rates.
