# Supplementary material for: Restriction Endonucleases that Bridge and Excise Two Recognition Sites from DNA
Source: J Mol Biol. 2007 Mar 23;367(2):419–31. doi: 10.1016/j.jmb.2006.12.070 (PMC1892151; doi:10.1016/j.jmb.2006.12.070)
Supplement: Supplementary Figures S1–S3 [file mmc1.pdf]

Submission to Journal of Molecular Biology.

Supplementary Data for -

Restriction Endonucleases that Bridge Two Recognition Sites and Cleave Eight Phosphodiester Bonds

Jacqueline J. T. Marshall, Darren M. Gowers, and Stephen E. Halford *

The DNA-Protein Interactions Unit, Department of Biochemistry, School of Medical Sciences, University of Bristol, University Walk, Bristol BS8 1TD, UK

* Corresponding author:
Phone: +44-(0)117-928-7429
FAX: +44-(0)117-928-8274
E-mail: s.halford@bristol.ac.uk

Supplementary Figure S1

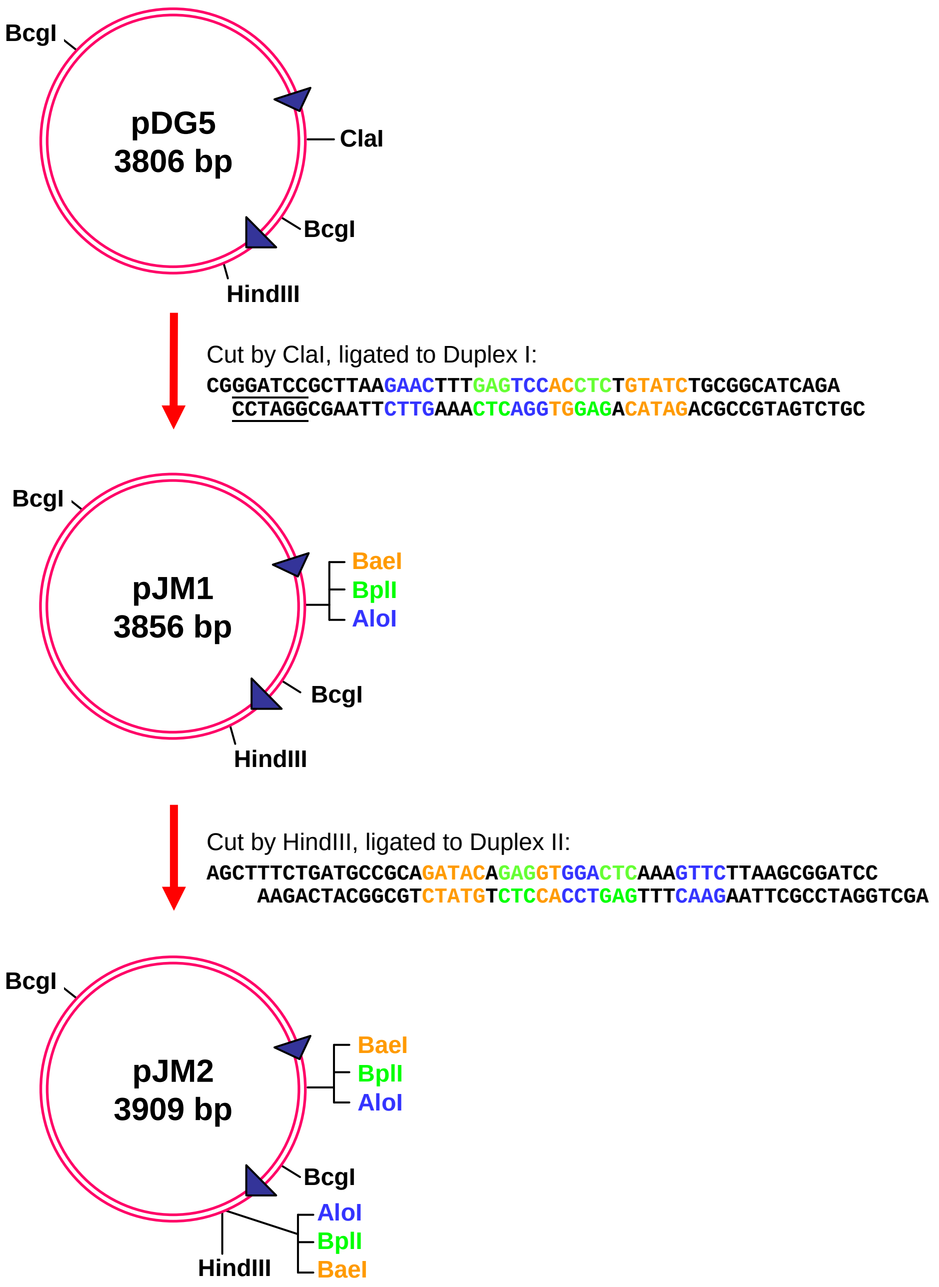

Construction of plasmids containing one or two AloI, BaeI and BplI recognition sites

The plasmid PDG5 was cleaved at its single ClaI site and the linear product ligated to Duplex I, which contains recognition sequences for AloI, BaeI and BplI (marked in blue, orange and green, respectively) and for BamHI (underlined). The ligation yielded the plasmid pJM1, which in turn was cleaved at its single HindIII site and ligated to Duplex II. Duplex II has the same 5′–3′ sequence as Duplex I and so also carries recognition sites for AloI, BaeI and BplI (coloured as above) within the same sequence context as Duplex I. The ligation yielded, among others, the plasmid pJM2, that had two sites for AloI, for BaeI and for BplI, in the orientation shown. All of the plasmids shown also carry two *res* sites from Tn*21* in repeated orientation (noted as blue arrowheads): in the catenane derived from pJM2, each ring has one site for AloI, for BaeI and for BplI.

Supplementary Figure S2

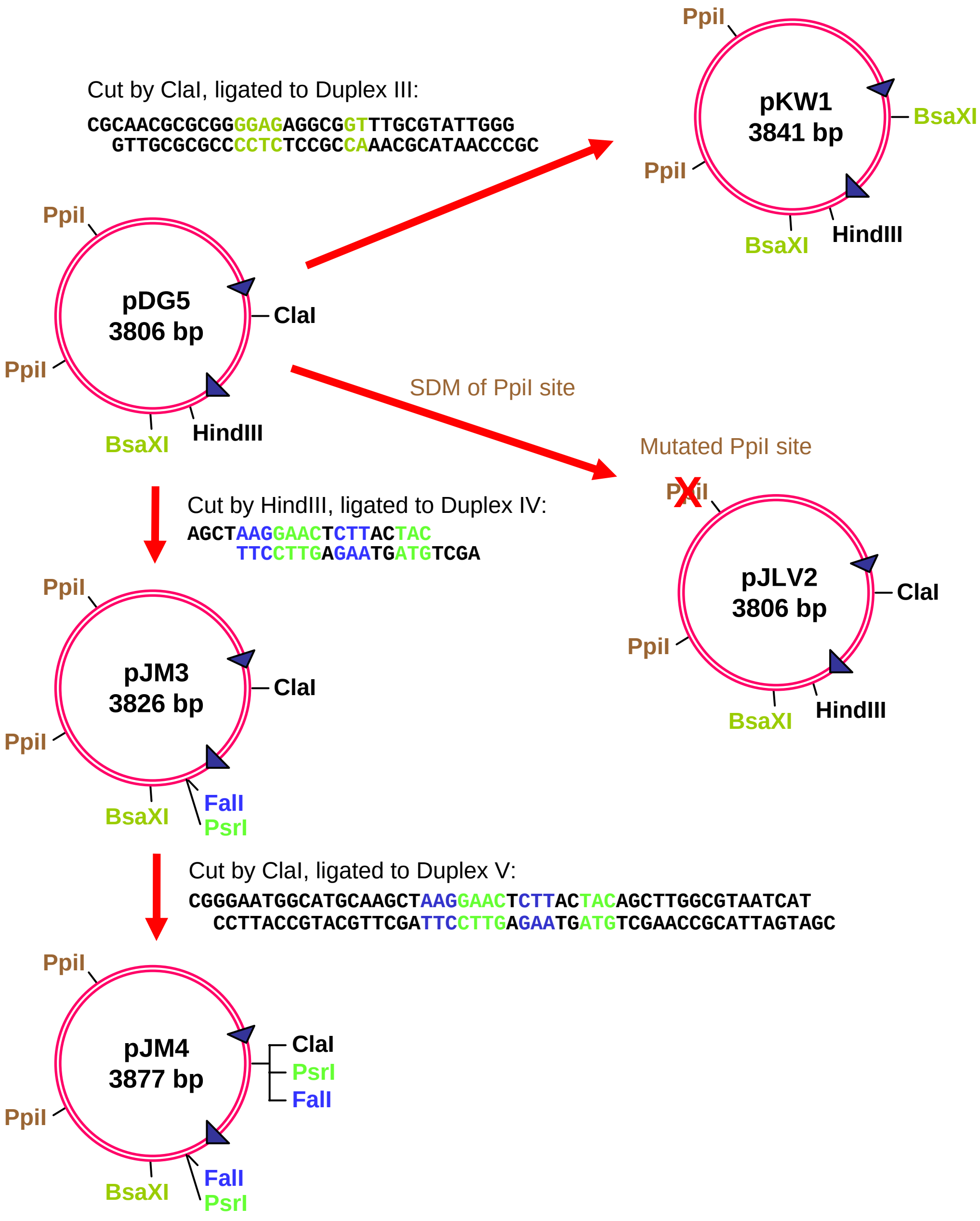

Construction of plasmids containing one or two PpiI, BsaXI, FalI and PsrI recognition sites

The plasmid pDG5 was cleaved at its ClaI site and the linear product ligated to Duplex III, which carries the recognition sequence for BsaXI (in olive), to give the plasmid, pKW1, with two recognition sites for BsaXI in inverted orientation. The initial plasmid pDG5 has two (inverted) PpiI sites: a plasmid with a single PpiI site, pJLV2, was constructed by using site-directed mutagenesis (Stratagene QuikChange) to make two single-base changes, one in each part of the bipartite recognition sequence, in one of the PpiI sites of pDG5 (as indicated by the red cross). Plasmids with one and with two (inversely-oriented) recognition sites for FalI and PsrI were also constructed from pDG5: by first cutting pDG5 with HindIII and ligating it to Duplex IV, to give pJM3; then by cutting pJM4 with ClaI and ligating it to Duplex V, to give pJM4. Duplexes IV and V both contain recognition sites for FalI and PsrI (in blue and green respectively): the sequences around these sites in Duplex V are the same as those in pJM3. All of the plasmids shown also carry two res sites from Tn21 in directly repeated orientation (noted as blue arrowheads).

Supplementary Figure S3

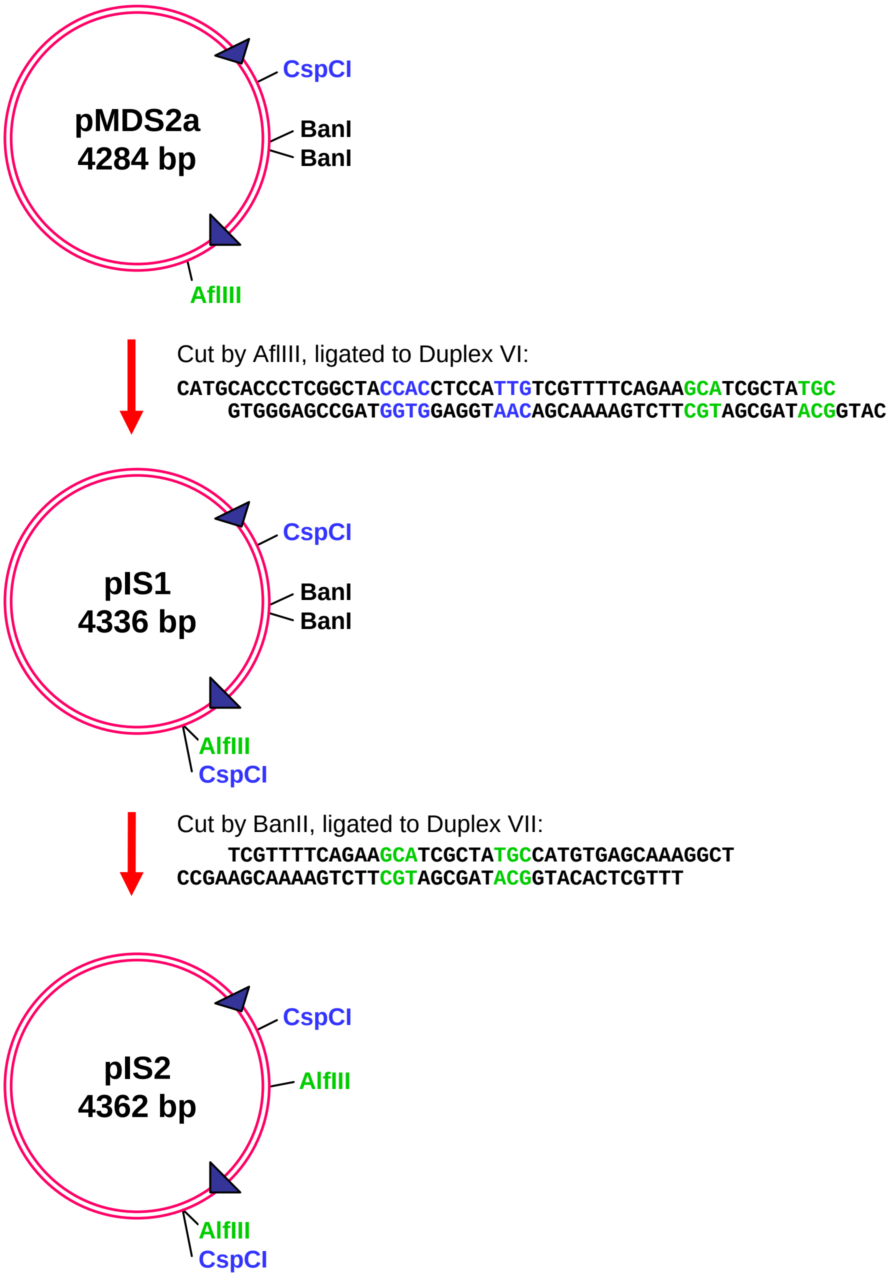

Construction of plasmids containing one or two CspCI and AlfI recognition sites

The plasmid pMDS2a was cleaved at its single AflI site and the linear product ligated to Duplex VI, which contains recognition sequences for CspCI and AlfI (marked in blue and green, respectively): the CspCI site in the duplex is in the same sequence context as the native site in pMDS2a. The ligation yielded the plasmid pIS1, which has two CspCI sites in inverted orientation and one AlfI site. It in turn was cleaved with BanII and ligated to Duplex VII. Duplex VII carries the recognition sequence for AlfI (in green), within the same sequence context as the AlfI site in pIS1. The ligation yielded, among others, the plasmid pIS2, that has two inversely oriented AlfI sites. All of the plasmids shown also carry two *res* sites from Tn*21* in repeated orientation (noted as blue arrowheads): in the catenanes derived from pIS1 and pIS2, each ring carries one CspCI site.
